# Sex as a predictor and moderator of psychosocial determinants of cardiometabolic risks for Métis People in Canada

**DOI:** 10.3389/fstro.2026.1736530

**Published:** 2026-02-12

**Authors:** Shara Johnson, Samantha Moore, Muqtasida Fatima, Adam McInnes, Heather Foulds

**Affiliations:** 1 College of Kinesiology, University of Saskatchewan, Saskatoon, SK, Canada; 2 Member of Saskatoon Métis Local 126, Citizen of Métis Nation-Saskatchewan, Saskatoon, SK, Canada; 3 College of Engineering, University of Saskatchewan, Saskatoon, SK, Canada

**Keywords:** cardiometabolic risks, cardiovascular disease, Indigenous Peoples, psychosocial stressors, sex differences, stroke

## Abstract

**Introduction:**

Métis People, one Indigenous group in Canada, have distinct culture, identity, and experiences. The determinants of Métis People's health, including stroke risk, may differ from other groups. This study examined relationships between psychosocial and lifestyle factors with cardiometabolic risk, a stroke risk indicator, and the moderating role of sex among Métis adults living in Saskatchewan, Canada.

**Methods:**

A community-based cross-sectional observation study was conducted with 70 Métis adults (39 ± 16 years; 66% female). Hierarchical multiple regression, moderated by sex, assessed psychosocial and lifestyle predictors of cardiometabolic risks (blood cholesterol, fasting glucose, glycosylated hemoglobin, blood pressure, and waist circumference).

**Results:**

Psychosocial factors were significantly associated with cardiometabolic risk components. Psychological distress, adverse childhood experiences, age and sex explained 30% of the variance in average blood pressure, *F* (4, 65) = 6.997, *p* = 0.01. Well-being, discrimination experiences, age and sex significantly explained 27% of the variance in average blood pressure, *F* (4, 65) = 5.89, *p* = 0.04. Sex moderated relationships of wellbeing with glycosylated hemoglobin, *F* (6, 63) = 7.374, *p* = 0.02, *R*^2^ = 0.414, with age (β = −0.497, *p* < 0.01), wellbeing (β = 0.593, *p* = 0.01), and wellbeing × sex (β = −0.53, *p* = 0.01) being significant predictors. Psychological distress moderated by sex also predicted total cholesterol. Lifestyle factors did not significantly predict cardiometabolic risk.

**Discussion:**

Psychosocial determinants, particularly wellbeing, play a central role in Métis People's cardiometabolic risk, with effects differing by sex. This emphasized the need for Métis-specific, trauma, and sex-informed approaches to cardiovascular health promotion.

## 1 Introduction

Canada has three recognized Indigenous groups, First Nation, Métis, and Inuit, with each group having distinct culture, identity, traditions, and experiences (Statistics Canada, 2022). Métis People, one of the fastest growing groups in Canada, have unique health experiences, grounded in strong kinship ties, resilience, and self-determination (Statistics Canada, 2021; Foulds et al., 2025; Ginn et al., 2025). Understanding Métis People's health therefore requires approaches that are culturally grounded and considers Métis People's unique historical and contemporary experiences.

Stroke is a leading cause of death in Canada; however, Indigenous Peoples are disproportionately and inequitably affected (Statistics Canada, 2025; Heart and Stroke, 2025). Although data specific to Métis People is limited, available evidence suggests higher rates of chronic health conditions, including cardiovascular diseases (CVD) such as hypertension, heart disease, and stroke compared to non-Indigenous People, with patterns differing from First Nations Peoples (Atzema et al., 2015; Martens et al., 2011; Statistics Canada, 2009; Foulds et al., 2013). The stroke mortality risk is twice as high for Indigenous Peoples than non-Indigenous Peoples Heart and Stroke, 2025. One research study examining the cardiovascular (CV) health of Métis People living in Ontario has shown a 25% greater prevalence of stroke in Métis People compared to the general population (Atzema et al., 2015). Sex and gender differences in the incidence of CVD are also evident, with Métis females in Alberta experiencing 37% greater ischemic heart disease incidence, while Métis males experience 24% greater incidence than non-Indigenous Peoples (Randall et al., 2019).

Determinants of CVD vary, and include both lifestyle and traditional factors (Liu et al., 2006). Lifestyle or behavioral risk factors include physical inactivity and sedentarism, tobacco smoking, and high carbohydrate intake (Liu et al., 2006). While the risks of CVD have declined over time among the general populations through lifestyle changes, the risks among Indigenous Peoples have remained stable and, in some instances, increased (Aziz et al., 2022; Reading, 2015). Factors including historic and ongoing trauma, food insecurity, and high levels of physical inactivity disproportionately impact Indigenous Peoples health with the root causes linked to colonization (Reading, 2009; Ironside et al., 2021; Barton et al., 2005). Acute and chronic stress, including traumatic life events, have long been identified as predictors of CVD in the general population (Humphries et al., 2017). Like other Indigenous groups, colonization has resulted in greater inequities in these risk factors for Métis People when compared to the general population (Reading and Wien, 2009).

Traditional CV risk factors include age, hypertension, and a cluster of metabolic indicators such as blood cholesterol and fasting glucose (Foulds et al., 2013; Cornier et al., 2008; Arnett et al., 2019). However, these factors considered in isolation only reflect a fraction of the total risk, particularly across diverse populations (Arnett et al., 2019). Composite cardiometabolic risk (CMR) indices, which integrate metabolic and CV indicators, offer a comprehensive assessment of an individual's risk profile (Cui et al., 2025; Satapathy et al., 2025; Foulds et al., 2013; Kulak et al., 2024). Established CMR measures typically include indicators such as cholesterol, high-density lipoprotein (HDL), fasting glucose, triglycerides, glycosylated hemoglobin (A1C), waist circumference, and blood pressure (Foulds et al., 2013; Leiter et al., 2011). CMR is strongly associated with stroke and other CV outcomes (Cui et al., 2025; Satapathy et al., 2025) and has been linked to other psychosocial stressors including discrimination and internalized racism among racialized populations (Tull et al., 2007; McKinley et al., 2020). However, these relationships remain unclear for Métis People. The objective of this study, therefore, is to examine the relationships between psychosocial and lifestyle factors with CMR and the moderating role of sex in these relationships among Métis People.

## 2 Materials and methods

### 2.1 Ethical considerations and participants

This Métis community-based cross-sectional observation study took place in the province of Saskatchewan, Canada. Saskatchewan is part of the Homeland of Métis People and has one of the highest proportions of Métis People, comprising approximately 6% of the population, and is projected to grow faster than the non-Indigenous population (Statistics Canada, 2022, 2021; Gaudry, 2009). This research was conducted in partnership with Saskatoon Métis Local 126, a local-level organization of Métis government in Saskatchewan, and aimed to prioritize community needs and self-determination, and to ensure Indigenous research sovereignty. The research team, which included Métis individuals and a Community Advisory Committee (CAC), worked collectively to ensure community voices were centered throughout, including co-designing the research, selecting assessment measures, and assisting with recruitment. Ethical approval was obtained from both Saskatoon Métis Local 126 and the University of Saskatchewan Biomedical Research Ethics Board. Eligible participants were at least 18 years of age, self-identified as Métis, descended from the historic Métis Nation, and were not pregnant.

### 2.2 Data gathering

Data gathering occurred in person between February 2023 and August 2025 at the Merlis Belsher Place, University of Saskatchewan. Information regarding participants' age, sex assigned at birth, gender identity (woman, man, gender-fluid), Métis community affiliation, education, and employment status was collected via questionnaires. Information regarding health determinants including psychosocial and lifestyle factors was gathered using validated and culturally adapted questionnaires. Determinants examined were identified using suggestions from the advisory committee and findings from the first phase of the larger project (Foulds et al., 2025). Psychosocial health determinants examined included psychological distress assessed using the Kessler Psychological Distress Scale (K10), a 10-item questionnaire about emotional state including anxiety and depressive symptoms a person experienced (Kessler et al., 2002). Responses were scored from 0 to 4 and them summed, with higher scores indicating greater psychological distress (Kessler et al., 2002). This scale has previously been used with Indigenous populations in Canada (Hajizadeh et al., 2021). Internal consistency for this scale had strong reliability (Cronbach's α = 0.86). The WHO-5 Well-Being Index, which comprises five questions, was used to assess positive moods, energy, and interests (Löwe et al., 2004). Responses were scored from 0 to 5 and then summed, with higher scores indicating greater wellbeing. Internal consistency for this scale had strong reliability (Cronbach's α = 0.82). Exposure to trauma was assessed by the 10-item Adverse Childhood Experience (ACE) questionnaire that assessed experiences with abuse and household dysfunction during childhood (Felitti et al., 2019). Responses were scored from 0 to 1 and summed, with higher scores indicating greater adverse childhood experiences. Internal consistency for this scale had strong reliability (Cronbach's α = 0.81). Discrimination experience was assessed by four questions representing the dimensions of perceived community racism, lifetime discrimination, everyday discrimination, and racism as a barrier to PA (Williams et al., 1997; Edwards and Cunningham, 2013; Williams, 1999; Ironside et al., 2024). Responses were scored from 0 to 2 and then summed, with higher scores indicating greater discrimination experiences. Internal consistency for this scale had acceptable reliability (Cronbach's α = 0.60).

Lifestyle factors assessed included diet and physical activity levels. Physical activity was assessed using the standardized Global Physical Activity Questionnaire (GPAQ), developed by the WHO to assess quantity (metabolic expenditure; METS) and domain (work, transport, and leisure-time) PA, in cross-cultural settings (Herrmann et al., 2013). The GPAQ has been found to be a useful measure for PA in Indigenous populations because it can be adapted to be specific to the culture and contexts (Esgin et al., 2021). Like all the other questionnaires, this was reviewed by the CAC and modifications were made to make it specific to Métis People living in Canada. Modifications included identifying the traditional Métis physical activities and activities specific to the physical environment, e.g., Red River jigging, snow shoveling, large-game hunting, and lacrosse. Responses from the GPAQ were converted to MET minutes per week, with 4 METS representing moderate PA, and 8 METS representing vigorous PA (Amelina, 2025). Internal consistency for this scale had acceptable reliability (Cronbach's α = 0.76). Assessment for a balanced healthy diet was done using the Mediterranean Eating Pattern for Americans (MEPA; Cerwinske et al., 2017). There is evidence supporting the validity of Mediterranean diet to Indigenous populations (Bourne et al., 2024; Parry-Strong et al., 2023). The MEPA is a 16-item questionnaire with responses scored from 0 to 1 and then summed, with higher scores indicating a diet more consistent with a Mediterranean pattern. Internal consistency for this scale had acceptable reliability (Cronbach's α = 0.70).

Cardiometabolic risk was assessed in a single in-laboratory visit after an overnight fast or at least 10 h of fasting. Measures included blood cholesterol and glucose levels, systolic and diastolic blood pressure (SBP, DBP), weight, height, and waist circumference. Point-of-care devices provided metabolic results within 7 min from a single 40 μl finger-sample drop of blood, conducted by a trained technician. The Cholestech LDX 3.41 machine (Alere Inc., San Diego, CA, United States) measured total cholesterol; HDL; low-density lipoprotein cholesterol (LDL), and triglyceride cholesterol and fasting glucose. A DCA Vantage Analyzer (Siemens Healthineers, Norwood, MA) measured A1C from 1 μl of blood. A composite measure for CMR was created using the standardized scores (*z*-scores) of waist circumference, average of SBP and DBP combined, fasting blood glucose, triglycerides, negative HDL, total cholesterol, and A1C (Kulak et al., 2024; Henry et al., 2009; Whitaker et al., 2018). Equal weights were used to create the CMR because there is currently no validated, population-specific weighting for CMR among Métis People, and externally derived weights from the general population may not be contextually or culturally appropriate. As HDL is a health protective variable, HDL scores were reverse coded so that higher values represented greater risk (Henry et al., 2009; Whitaker et al., 2018). Sensitivity analyses showed that the findings were robust to treatment of HDL.

### 2.3 Statistical analysis

Data analysis was performed using Statistical Package for the Social Sciences (SPSS) Version 28.0.1.0 (IBM Corp., Armonk, NY, United States). Descriptive statistics were used to summarize participants' characteristics and psychosocial, lifestyle, and CMR variables, with categorical variables represented by frequencies and percentages, and continuous variables by means and standard deviations (SD). In this study, sex refers to biological sex assigned at birth and was coded as 0 = male, 1 = female to facilitate analyses in SPSS. Gender identity was collected but not analyzed due to the small number of gender-diverse participants (*n* = 2), which posed a confidentiality risk. All gender-diverse participants reported to be of female sex at birth; therefore, sex-based analyses combined females and gender-diverse individuals. Sex differences in means were examined by independent-sample *t-*tests. Pearson's *r* correlation was used to examine the intercorrelation among the predictor and outcome variables. A series of hierarchical multilinear regression analyses was used to examine relationships of psychosocial variables and lifestyle factors with composite and individual components of CMR, while controlling for age, with sex included as both a predictor and a moderator in all models. Using G^*^Power sample size analysis and Cohen (1988) statistical power guidelines, a sample size of 68 was calculated to be sufficient to detect moderate effect sizes **(***f*^2^ = 0.15) in multiple linear regression models with an alpha level of 0.05, a power of 0.80, and up to six predictor variables and covariates (Faul et al., 2009). To reduce model overfitting and preserve statistical power, predictors were grouped into conceptually related blocks: (1) psychological distress and adverse childhood; (2) wellbeing and discrimination; and (3) diet and physical activity. Data collection was done in person with the assistance of two members of the research team, resulting in minimal missing data, which was addressed using pairwise deletion. All regression assumptions of linearity, multicollinearity, normality, and homoscedasticity were met.

## 3 Results

Table 1 gives a demographic overview of the participants. The majority of the participants were females (66%), and all the participants who identified as males also identified as men. *t*-test examinations revealed that age, body mass index, waist circumference, SBP, DBP, and CMR factors, except for HDL, were not statistically different between males and females. Métis females had higher HDL levels than males. Bivariate correlations (Table 2) indicated significant correlations of metabolic variables with age, justifying the inclusion of age as a covariate in models.

**Table 1:** Demographic characteristics of Métis adults in Saskatchewan by sex and overall participant group, *N* (%), mean ± SD.

**Characteristic**	**Female participants (*n* = 46)**	**Male participants (*n* = 24)**	**Overall participants (*n* = 70)**	**Missing data (%)**
**Gender**				
Woman	44 (96)	0	44 (63)	
Man	0	24 (100)	24 (34)	
Gender-fluid	2 (4)	0	2 (3)	
Age (years)	40 ± 17	38 ± 17	39 ± 16	
**Education**				
High school	8 (17)	5 (21)	13 (18)	
Vocational school	18 (39)	8 (33)	26 (37)	
College/university	11 (24)	7 (29)	18 (26)	
Professional/graduate school	9 (20)	4 (17)	13 (19)	
**Grew up in traditional community**				
Yes	10 (22)	2 (8)	12 (17)	
No	29 (63)	19 (79)	48 (69)	
Some	7 (15)	3 (13)	10 (14)	
**Relocated from home community**				
Yes	25 (54)	10 (42)	35 (50)	
No	16 (35)	11 (46)	27 (39)	
N/A	5 (11)	3 (12)	8 (11)	
**Job permanence**				
Permanent	25 (54)	14 (58)	39 (56)	
Not permanent	17 (37)	9 (38)	26 (37)	
Other	4 (8)	1 (4)	5 (7)	
BMI (kg/m^2^)	28.53 ± 7.48	27.60 ± 2.88	27.6 ± 2.88	0.0
SBP (mmHg)	115.87 ±13.78	121.29 ± 12.32	117.73 ± 13.46	0.0
DBP (mmHg)	71.97 ±8.34	76 ± 9.22	73.35 ± 8.79	0.0
Waist circumference (cm)	95.19 ± 18.03	95.05 ± 9.06	95.15 ± 15.47	0.0
HDL (mmol/L)	1.49 ± 0.45	1.21 ± 0.38	1.39 ± 0.44	1.4
LDL (mmol/L)	2.42 ± 1.20	2.61 ± 0.93	2.48 ± 1.11	1.4
Triglycerides (mmol/L)	1.56 ± 1.33	1.19 ± 0.81	1.43 ± 1.18	0.0
Total cholesterol (mmol/L)	4.77 ± 1.22	4.47 ± 1.04	4.66 ± 1.16	0.0
Glucose (mmol/L)	5.09 ± 0.48	5.09 ± 0.29	5.09 ± 0.42	1.4
A1C (%)	5.41 ± 0.39	5.43 ± 0.40	5.42 ± 0.39	0.0
MET (min)	3,065.91 ± 4,590.58	7,319.67 ± 12,842.45	4,524.34 ± 8,535.55	0.0
Diet	6.73 ± 1.68	6.83 ± 1.95	6.77 ± 1.77	1.4
Discrimination	4.35 ± 2.21	3.79 ± 1.67	4.16 ± 2.20	0.0
Psych. distress	24.52 ± 5.74	22.04 ± 7.30	23.67 ± 6.38	0.0
Wellbeing	13.98 ± 4.45	14.42 ± 3.74	14.13 ± 4.19	0.0
ACE	3.26 ± 2.70	3.08 ± 2.83	3.20 ± 2.73	0.0

A1C, glycosylated hemoglobin; ACE, adverse childhood experiences; BMI, body mass index; DBP, diastolic blood pressure; HDL, high-density lipoprotein; LDL, low-density lipoprotein; MET, metabolic equivalents; N, number of participants; SBP, systolic blood pressure; SD, standard deviation.

**Table 2:** Means, standard deviations, and intercorrelations among psychosocial, lifestyle, and metabolic variables of Métis adults in Saskatchewan (*N* = 70).

**Variable**	**Age**	**MET**	**Diet**	**Discr**.	**Psych. distress**	**Wellbeing**	**ACE**	**Waist**	**Chol**	**A1C**	**BP**	**CMR**
Age	1.00											
MET	−0.07	1.00										
Diet	0.12	−0.02	1.00									
Discrimination	0.08	0.05	0.05	1.00								
Psych. distress	−0.38^**^	−0.06	−0.16	0.40^**^	1.00							
Wellbeing	0.21	0.15	0.01	−0.29^*^	−0.57^**^	1.00						
ACE	−0.02	−0.05	−0.10	0.55^**^	0.31^**^	−0.31^**^	1.00					
Waist	0.39^**^	−0.11	0.00	0.20	−0.03	−0.12	0.22	1.00				
Cholesterol	0.31^**^	−0.08	0.14	−0.05	−0.10	−0.01	0.13	0.17	1.00			
A1C	0.56^**^	0.00	0.13	0.12	−0.27^*^	0.20	−0.05	0.43^**^	0.08	1.00		
Average BP	0.42^**^	0.07	0.09	0.17	−0.01	−0.05	0.20	0.52^**^	0.19	0.26^*^	1.00	
CMR	0.55^**^	−0.09	0.15	0.09	−0.10	0.03	0.14	0.78^**^	0.37^**^	0.64^**^	0.70^**^	1.00

ACE, adverse childhood experiences; A1C, glycosylated hemoglobin; BP, blood pressure; CMR, cardiometabolic risk; Discr., discrimination; MET, metabolic equivalents; N, number of participants; SD, standard deviation.

^*^p < 0.05.

^**^p < 0.01.

### 3.1 Psychosocial and lifestyle factors with composite CMR

Table 3 gives a summary of the results for hierarchical models that examined the relationships of psychosocial and lifestyle factors with overall CMR. In examining the role of sex in moderating the relationships between psychological distress and adverse childhood experiences with CMR, step 1, age entered by itself, accounted for 31% of the variance in CMR, *F* (1, 65) = 28.519, *p* < 0.001. In step 2, the addition of the main effects of psychological distress, adverse childhood experiences, and sex did not significantly improve the predictability of the model. The addition of interaction terms (psychological distress × sex, and adverse childhood experiences × sex) in the third step also did not significantly enhance the predictive value of the model.

**Table 3:** Hierarchical regression models for relationships of composite CMR with psychosocial and lifestyle factors in Métis adults in Saskatchewan.

**Adverse childhood experiences and psychological distress**						**Discrimination and wellbeing**					
**Predictor**	**R** ^2^	Δ**R**^2^	Δ**F**	β	**t**	**Predictor**	**R** ^2^	Δ**R**^2^	Δ**F**	β	**t**
Step 1	0.31	0.31	28.52^***^			Step 1	0.31	0.31	28.52^***^		
Age				0.56	5.34^***^	Age				0.55	5.34^***^
Step 2	0.37	0.06	1.92			Step 2	0.34	0.03	0.99		
Age				0.62	5.57^***^	Age				0.58	5.49^***^
Sex				−0.17	−1.66	Sex				−0.15	−1.47
Psych. distress				0.13	1.11	Wellbeing				−0.09	−0.76
ACE				0.12	1.10	Discr.				0.05	0.46
Step 3	0.37	0.01	0.27			Step 3	0.39	0.05	2.55		
Age				0.62	5.46^***^	Age				0.55	5.34^**^
Sex				−0.23	−1.26	Sex				−0.14	−1.32
Psych. distress				0.06	0.34	Wellbeing				0.13	0.59
ACE				0.09	0.49	Discr.				−0.16	−0.88
ACE × sex				0.08	0.45	Wellbeing × sex				−0.24	−1.18
Psych. distress × sex				0.09	0.39	Discr. × sex				0.27	1.47
**Diet and MET**											
**Predictor**	**R** ^2^	Δ**R**^2^	Δ**F**	β	**t**						
Step 1	0.30	0.30	27.12^***^								
Age				0.54	5.13^***^						
Step 2	0.33	0.03	0.96								
Age				0.55	5.11^***^						
Sex				−0.16	−1.44						
Diet				−0.08	−0.72						
MET				0.08	0.79						
Step 3	0.36	0.02	1.08								
Age				0.55	5.11^***^						
Sex				−0.17	−1.54						
Diet				−0.06	−0.47						
MET				−0.07	−0.47						
Diet × sex				−0.08	−0.65						
MET × sex				0.22	1.37						

Sex coded as 0 = male, 1 = female.

ACE, adverse childhood experiences; CMR, cardiometabolic risk; Discr., discrimination experiences; MET, metabolic equivalents.

^**^p < 0.01.

^***^p < 0.001.

In examining the moderating role of sex in the relationships between wellbeing and discrimination experiences, in step 1, age entered by itself, explained 31% of the variance in overall CMR, *F* (1, 65) = 28.519, *p* < 0.001. However, the addition of the main effects of wellbeing, discrimination experiences, and sex in step 2 did not improve the predictability of the model. Likewise, for step 3, the variance explained by adding the interaction terms (general wellbeing × sex, and discrimination × sex) did not increase significantly, Δ*R*^2^ = 0.052, Δ*F* (2, 60) = 2.547, *p* = 0.087.

The regression models assessing the relationships between the lifestyle factors diet and physical activity levels with composite CMR did not show diet and physical activity to be significant predictors of CMR. In step 1, with age only entered, age accounted for 30% of the variance in CMR, *F* (1, 64) = 27.118, *p* < 0.001. Adding the main effects of diet, physical activity, and sex in step 2 did not significantly improve the variance explained. The addition of the interaction terms (diet × sex, and physical activity × sex) in step 3 also did not improve the variance explained in CMR.

### 3.2 Psychosocial factors and components of CMR

Table 4 gives a summary of the significant findings for hierarchical models that examined the relationships of psychosocial and lifestyle factors with individual components of CMR (waist circumference, average blood pressure, fasting blood glucose, triglycerides, HDL, total cholesterol, and A1C). In examining average blood pressure, age was entered in step 1 of the hierarchical regression and accounted for 17% of the variance in average blood pressure, *F* (1, 68) = 13.532, *p* < 0.001. In step 2, the addition of the main effects of psychological distress, adverse childhood experiences, and sex significantly predicted average blood pressure, increasing the variance explained to 30%, *F* (4, 65) = 6.997, *p* = 0.01, indicating that the model was a reliable predictor of average blood pressure levels. Age (β = 0.49, *p* < 0.001) and sex (β = −0.29, *p* = 0.01) were significant contributors to the model. However, in step 3, the addition of the interaction terms (psychological distress × sex, and adverse childhood experiences × sex) did not result in an increase in the variance, suggesting that the relationships were not moderated by sex.

**Table 4:** Hierarchical regression models for relationships of CMR components with psychosocial and lifestyle factors in Métis adults in Saskatchewan.

**ACE, psychological distress, and cholesterol**						**ACE, psychological distress, and average BP**					
**Predictor**	**R** ^2^	Δ**R**^2^	Δ**F**	β	**t**	**Predictor**	**R** ^2^	Δ**R**^2^	Δ**F**	β	**t**
Step 1	0.09	0.09	7.33^**^			Step 1	0.17	0.17	13.53^***^	
Age				0.31	2.71^**^	Age				0.41	3.68^***^
Step 2	0.13	0.03	0.80			Step 2	0.30	0.14	4.19^*^	
Age				2.20	2.26^*^	Age				0.49	4.30^***^
Sex				0.11	0.95	Sex				−0.29	−2.69^**^
Psych. distress				−0.05	−0.39	Psych. distress				0.16	1.33
ACE				0.15	1.25	ACE				0.19	1.70
Step 3	0.24	0.11	4.42^**^			Step 3	0.31	0.01	0.19	
Age				0.28	2.28^*^	Age				0.49	4.27^***^
Sex				−0.16	−0.81	Sex				−0.20	−1.08
Psych. distress				−0.27	−1.42	Psych. distress				0.23	1.29
ACE				−0.13	−0.64	ACE				0.20	1.07
ACE × sex				0.34	1.76	ACE × sex				−0.02	−0.09
Psych. distress × sex				0.43	1.70	Psych. distress × sex				−0.14	−0.56
**Discrimination, wellbeing, and A1C**						**Discrimination, wellbeing, and average BP**					
**Predictor**	**R** ^2^	Δ**R**^2^	Δ**F**	β	**t**	**Predictor**	**R** ^2^	Δ**R**^2^	Δ**F**	β	**t**
Step 1	0.32	0.32	31.42^***^			Step 1	0.17	0.17	13.53^***^		
Age				0.56	5.48^***^	Age				0.41	3.68^***^
Step 2	0.34	0.02	0.75			Step 2	0.27	0.10	2.96^*^		
Age				0.53	5.09^***^	Age				0.44	4.00^***^
Sex				−0.06	−0.63	Sex				−0.27	−2.52^*^
Wellbeing				0.12	1.14	Wellbeing				−0.13	−1.09
Discr.				0.12	1.12	Discr.				0.12	1.09
Step 3	0.41	0.07	3.94^*^			Step 3	0.29	0.03	1.17		
Age				0.49	4.92^**^	Age				0.45	4.08^***^
Sex				−0.05	−0.53	Sex				−0.26	−3.09^*^
Wellbeing				0.59	2.92^**^	Wellbeing				−0.19	−0.84
Discr.				0.14	0.79	Discr.				−0.12	−0.63
Wellbeing × sex				−0.53	−2.68^*^	Wellbeing × sex				0.07	0.32

Sex coded as 0 = male, 1 = female.

ACE, adverse childhood experiences; A1C, glycosylated hemoglobin; BP, blood pressure; CMR, cardiometabolic risk; Discr., discrimination experiences.

^*^p < 0.05.

^**^p < 0.01.

^***^p < 0.001.

In examining total cholesterol, step 1, age entered by itself, accounted for 10% of the variance in total cholesterol, *F* (1, 68) = 7.333, *p* < 0.001. In step 2, the main effects of psychological distress, adverse childhood experiences, age, and sex did not significantly predict total cholesterol, indicating that the model was not a reliable predictor of total cholesterol. However, in step 3, the interaction terms (psychological distress × sex, and adverse childhood experiences × sex) were added, and the variance explained increased significantly to 24%, *F* (6, 63) = 3.255, *p* = 0.01, suggesting that the interaction contributed unique explanatory value beyond the main effects. Within this model, age emerged as a significant predictor (β = 0.277, *p* = 0.026).

Controlling for age, there were no significant findings for the models examining the relationships between psychological distress, adverse childhood experiences, and the components of waist circumference, HDL, glucose, triglycerides, and A1C.

To examine whether sex moderated the relationship of wellbeing and discrimination with A1C, age entered in the first step of the hierarchical regression accounted for 32% of the variance explained, *F* (1, 68) = 31.42, *p* < 0.01. The main effects of wellbeing, discrimination, and sex did not significantly predict A1C levels in step 2. In step 3, the interaction terms (wellbeing × sex, and discrimination × sex) were entered, and the change in variance explained significantly increased to 41%, *F* (6, 63) = 7.37, *p* = 0.02, suggesting that the interaction contributed unique explanatory value beyond the main effects. For model 3 in this regression, age (β = −0.49, *p* < 0.01), wellbeing (β = 0.59, *p* = 0.01), and the interaction term wellbeing × sex (β = −0.53, *p* = 0.01) were the significant predictors. This significant interaction suggests that the association between wellbeing and A1C differed by sex such that higher wellbeing was associated with higher A1C for males, but this association was weaker or absent in females.

To further evaluate the robustness of this interaction, sensitivity analyses were conducted using sex-stratified regression models. Among females, in the regression model examining the association of discrimination and wellbeing with A1C, controlling for age was not significant. However, among males the model was significant and explained 55% of the variance in A1C, *F* (3, 20) = 8.10, *p* < 0.001, Δ*R*^2^ = 0.20. Within the male subgroup, wellbeing was a significant predictor (β = 0.54, *p* = 0.01), indicating that higher wellbeing was associated with higher A1C. These findings corroborated the moderation results and support a sex-specific association between and A1C.

Examining the relationship of average blood pressure with wellbeing and discrimination, in step 1 with age only, the model accounted for 17% of the variance in average blood pressure, *F* (1, 68) = 13.53, *p* < 0.001. In step 2, the main effects of wellbeing, discrimination experiences, age, and sex significantly predicted average blood pressure, accounting for an increased variance to 27%, *F* (4, 65) = 5.89, *p* = 0.04, indicating that the model was a reliable predictor of average blood pressure levels, with age (β = 0.44, *p* < 0.001) and sex (β = −0.27, *p* = 0.01) being the significant contributors. However, the addition of the interaction terms (wellbeing × sex, and discrimination × sex) in step 3 did not result in an increase in the variance, suggesting that the relationships were not moderated by sex.

Controlling for age, there were no significant findings for the models examining the relationship of wellbeing, discrimination experiences, and the CMR components of waist circumference, HDL, glucose, and triglycerides.

There were also no significant findings for any of the models examining the relationship of lifestyle factors, diet, and physical activity, with any of the individual components of CMR, suggesting that for Métis People lifestyle factors alone did not significantly predict CMR.

## 4 Discussion

The objective of the study was to examine relationships between psychosocial and lifestyle factors with CMR and the moderating role of sex in these relationships among Métis adults living in Saskatchewan, Canada. This is the first known study to explore determinants of both composite and individual CMR components to provide a more nuanced examination of cardiometabolic pathways for Métis People. This significantly added to the limited evidence on Métis People's CV health. Several important findings emerged from this study, including the influential role of sex and psychosocial factors in CMR in Métis People.

When the individual components of CMR were examined, psychosocial factors were found to be significant predictors of specific CMR indicators. In particular, psychological distress and adverse childhood experiences, along with the covariates age and sex, significantly predicted average blood pressure. Additionally, average blood pressure was predicted by wellbeing and discrimination with the covariates age and sex in another model. These findings highlight the important role psychosocial stressors, both historical and contemporary, play in shaping blood pressure levels, and consequently hypertension risk among Métis People. Previous research has shown that in addition to being higher among Métis People compared to the general population, the prevalence of the overall burden of hypertension for Métis People has remained stable over time (Koprich et al., 2024). The present findings extend this body of evidence by providing Métis-specific insights into how cumulative and intergenerational exposure to psychosocial stressors such as childhood trauma, discrimination experiences, and the intergenerational impacts of colonization contribute to CMR in Métis People today (Lewis et al., 2021; Aguiar and Halseth, 2015; Phillips-Beck et al., 2019). Similar patterns have been observed in racialized groups where stress related to racial discrimination has also been linked to elevated levels of blood pressure and increased hypertension risk (Marwaha, 2022). The overall implication of our research findings is that psychosocial factors may contribute to the risk of stroke and other CVD in Métis People through their influence on hypertension. This emphasizes the need for Métis-specific health promotion initiatives that are trauma-informed and take a holistic approach to health that is geared toward identifying and effectively addressing the root causes of cardiometabolic health in Métis People.

Another significant finding of this study was that sex is both an independent predictor and a moderator of relationships of psychosocial factors with some of the CMR components. This research, the only known study to examine determinants of average blood pressure in Métis People, found that when age and psychosocial factors are controlled for, average blood pressure is lower in Métis females than in males. While previous research has indicated that average blood pressure was lower in First Nations females than males, this was not previously evidenced for Métis People (Foulds and Warburton, 2014). There are several possible explanations for these findings. First, in general, biological females tend to have lower blood pressure than males prior to menopause due to the hormonal differences (Ji et al., 2020). Given that the average age of the female participants in our study was 40 ± 17 years, these patterns might hold true for Métis females as well. Another explanation for these differences is that socially, Métis females may benefit from more social support than Métis males, which may serve as a buffer against psychosocial stressors (Ramage-Morin and Bougie, 2017). These findings from our study emphasize the need for health promotion initiatives that are gender sensitive, considering the role of both biological and psychosocial factors.

Another significant finding of our research is that the higher wellbeing scores in Métis males were associated with higher A1C levels. This counterintuitive finding suggests that perceived wellbeing may not consistently align with biological markers of metabolic health among Métis males. While prior research has generally identified wellbeing to be health protective in relation to CV risk, serving as a buffer against stressful situations, a motivator for positive health behaviors and promoting adaptive physical functioning (Sin, 2016), our findings indicate that this relationship may be more complex and context dependent. Several explanations may account for this pattern. Higher wellbeing among Métis males may reflect strong social connection, cultural engagement, and overall satisfaction with life, none of which necessarily correspond with favorable metabolic profiles. For example, social and cultural activities that enhance wellbeing may also involve dietary practices that influence glycemic regulation. Alternatively, individuals with fewer perceived health concerns may be less likely to engage in preventive health monitoring, resulting in unrecognized metabolic risk. Importantly, though A1C is a well-established indicator of ischemic stroke (Priya et al., 2024; Alhawiti et al., 2025), in this study A1C was examined as a continuous biomarker rather than a clinical diagnosis, and these findings should be cautiously interpreted. These findings, however, should be viewed as hypothesis-generating and require replication in larger samples, particularly among males. Overall, the implication of the finding is that health promotion initiatives for Métis People, particularly males, may benefit from integrating strengths-based approaches that support wellbeing while also emphasizing the value of routine metabolic screening and preventive care, regardless of perceived health status.

While significant relationships were observed between some individual CMR components and psychosocial factors, we did not find similar associations with the composite CMR used in our study. The CMR components included in our study are based on similar components used in studies with non-Indigenous populations (Kulak et al., 2024; Henry et al., 2009; Whitaker et al., 2018), and this suggests that factors to be included in creating a CMR risk profile for Métis People may differ from those of the general population. Our findings are supported by a review exploring heart health strategies for Indigenous Peoples, which found that the imbalanced reliance on biomedical risk factors for Indigenous Peoples has been problematic in addressing the underlying causes and outcomes of their heart health (Wali et al., 2024). The findings from our study indicate that a standard CMR may not adequately reflect the cultural, biological, and social distinctiveness of risk profiles for Métis People, potentially limiting the predictability for Métis Peoples. Cardiometabolic risk has been emerging as an effective assessment tool for predicting stroke and other CVD risks in other populations (Cui et al., 2025; Satapathy et al., 2025); therefore, it is important to identify the components that accurately capture the CMR profiles of Métis People. Furthermore, the non-significant findings for the composite CMR may also suggest that the equal weighting of the components used in this study may not fully reflect the risk profile for Métis People. Further research is needed to develop assessment tools that are culturally grounded and reflect Métis People's unique lived experiences and health determinants and identify the correct weightings for Métis People. This will improve the sensitivity of risk prediction and create more equitable health promotion strategies targeting cardiometabolic and overall health in Métis People.

Another important finding of this research was that the lifestyle factors of diet and physical activity did not significantly predict cardiometabolic health among Métis males and females. This result appears contradictory to extensive literature demonstrating positive associations between lifestyle behaviors and CMR in both the Indigenous and general populations (Foulds et al., 2013; McEligot et al., 2010; Lai et al., 2019). Conversely, these finding align with research identifying that general populations have reduced the prevalence of CVD through lifestyle change, while similar declines in CVD have not been experienced among Indigenous Peoples (Aziz et al., 2022). For example, studies have reported that physical activity levels for Métis People are similar or in some instances higher than for non-Indigenous populations (Findlay, 2011), yet Métis People remain disproportionately burdened by CVD (Martens et al., 2011; Foulds et al., 2013; Koprich et al., 2024). One possible explanation for this is that CMR is influenced by multiple intersecting determinants beyond lifestyle factors, including genetics, historical trauma, stress, access to healthcare, medication use, and socioeconomic conditions (Wali et al., 2024; Powell-Wiley et al., 2022). For Indigenous populations, colonial and structural determinants (e.g., intergenerational trauma, food insecurity, and systemic barriers to care) may overshadow or mediate the impact of lifestyle behaviors (Reading, 2009; Reading and Wien, 2009). This suggests that diet and physical activity may act as mediators, indirectly influencing CMR through physiological and psychological pathways. Future research is needed to explore the potential mediation effects. Another possible explanation for this non-significant finding is measurement limitation. The lifestyle questionnaires, adapted from tools designed for general populations, may lack sensitivity to capture meaningful differences for Métis People, highlighting the need for community-specific measures. Overall, these findings emphasize the need for health promotion strategies that move beyond individual behaviors to address societal and historical determinants, while also developing culturally relevant assessment tools.

Overall, the findings of this study filled a critical evidence gap in the determinants and risk factors of cardiometabolic health, and consequently stroke, for Métis People specifically. A major strength of this study is the inclusion of non-traditional determinants, including discrimination, adverse childhood experiences, psychological distress, and wellbeing to account for more culturally and socially relevant influences of Métis People's health beyond biomedical risk models. Furthermore, by examining both composite and individual CMR components, this study provides a more detailed understanding of the distinct cardiometabolic pathways influencing health among Métis People.

Despite these strengths, the study was not without limitations. First, the sample size, while adequate for exploratory analysis, constrained the number of variables that could have been included in the regression models without compromising statistical power, including interaction effects. This limited our ability to examine all psychosocial and lifestyle factors in a single model, control for additional potential confounders such as medication use and smoking history, and test for mediation effects such as for diet and physical activity. A larger sample would allow for more comprehensive modeling and robust conclusions.

Another limitation relates to the composite CMR measure used in this study. The CMR components were based on general population standards, which may not fully reflect the cultural, social, and biological distinctiveness of Métis People, potentially limiting predictive validity. Additionally, the lack of a community-specific weighting could have affected the measure's utility in identifying true risk patterns in this group. Future research can examine CMR components more comprehensively to identify and develop validated population-specific CMR assessment tools. Similarly, the questionnaires used to assess the psychosocial and lifestyle determinants were adapted from questionnaires that were initially created for the general population. Though these questionnaires were modified for our population and have been previously validated among Indigenous Peoples, they may still not adequately reflect Métis People's culture, experiences, and health realities. This further points to the need for more Métis People-specific assessment tools. Additionally, the use only of quantitative methods to explore this research also limits our understandings of some of the findings. Further research using qualitative methods, including interviews and other culturally relevant methods such as storytelling, can help to provide an in-depth and more comprehensive understanding of the relationships observed as well as uncover factors that may be significant to Métis People's CMR.

Another limitation is the examination of biological sex and not gender. While sex refers to biological attributes, gendered social experiences may influence Métis People's health outcome differently. In this study, participants' gender identity largely aligned with their biological sex except for two individuals. This suggests that gender identity alone may not fully capture gendered social experiences. Future research should incorporate broader dimensions of gender such roles or institutionalized gender norms; this may provide a more nuanced understanding of how gendered experiences affect Métis People's health.

## 5 Conclusion

This study highlighted both the complexity and distinctiveness of Métis People's CMR risks and CV health. The significant associations between the psychosocial factors including discrimination, adverse childhood experiences, and wellbeing, with individual CMR components emphasizing the influential role of social and historical determinants of Métis People's health. The predictive and moderating role of sex on individual CMR components illustrates that CMR in Métis People cannot be understood without considering sex and cultural contexts. Overall, these findings emphasize the need for Métis People-specific, culturally grounded health assessments and promotion initiatives that extend beyond behavioral models toward holistic, trauma-informed, and equity-oriented approaches.

## Data Availability

Data generated or analyzed during this study are not available due to the nature of this research as ethical approval and participant permission to share data was not obtained. The data are owned by Indigenous community partners outside of the research team and institution. Requests to access the datasets should be directed to heather.foulds@usask.ca.

## References

[B1] Aguiar W. Halseth R. (2015). Aboriginal Peoples and Historic Trauma: The Processes of Intergenerational Transmission. Prince George, BC: National Collaborating Centre for Aboriginal Health. Available online at: https://www.ccnsa-nccah.ca/docs/context/RPT-HistoricTrauma-IntergenTransmission-Aguiar-Halseth-EN.pdf (Accessed October 12, 2025)

[B2] Alhawiti N. M. Elsokkary E. M. Aldali J. A. Alotaibi B. A. (2025). Investigating the impact of glycated hemoglobin levels on stroke severity in patients with acute ischemic stroke. Sci. Rep. 15, 12114–12118. doi: 10.1038/s41598-025-95305-2 40204797 PMC11982240

[B3] Amelina R. (2025). Global Physical Activity Questionnaire Analysis Guide. Geneva: Surveillance and Population-Based Prevention Prevention of Noncommunicable Diseases Department World Health Organization. Available online at: https://www.who.int/docs/default-source/ncds/ncd-surveillance/gpaq-analysis-guide.pdf (Accessed July 1, 2025).

[B4] Arnett D. K. Blumenthal R. S. Albert M. A. Buroker A. B. Goldberger Z. D. Hahn E. J. . (2019). 2019 ACC/AHA guideline on the primary prevention of cardiovascular disease: a report of the American College of Cardiology/American Heart Association Task Force on clinical practice guidelines. J. Am. Coll. Cardiol. 74:e177. doi: 10.1016/j.jacc.2019.03.010 30894318 PMC7685565

[B5] Atzema C. L. Khan S. Lu H. Allard Y. E. Russell S. J. Gravelle M. R. . (2015). Cardiovascular disease rates, outcomes, and quality of care in Ontario Métis: a population-based cohort study. PLoS ONE 10:e0121779. doi: 10.1371/journal.pone.0121779 25793978 PMC4368556

[B6] Aziz H. Marchand M. Pop C. King A. Anand S. S. Arbour L. . (2022). A call to action: optimizing indigenous cardiovascular health in Canada. Can. J. Cardiol. 38, 1584–1587. doi: 10.1016/j.cjca.2022.06.011 35718241

[B7] Barton S. S. Anderson N. Thommasen H. V. (2005). The diabetes experiences of aboriginal people living in a rural Canadian community. Aust. J. Rural Health. 13, 242–246. doi: 10.1111/j.1440-1584.2005.00709.x 16048467

[B8] Bourne N. A. Gill G. Cooley K. (2024). Cultural adaptations addressing diversity and health access in the mediterranean diet: a realist synthesis. CAND J. 31, 29–36. doi: 10.54434/candj.146

[B9] Cerwinske L. A. Rasmussen H. E. Lipson S. Volgman A. S. Tangney C. C. (2017). Evaluation of a dietary screener: the Mediterranean Eating Pattern for Americans tool. J. Hum. Nutr. Diet. 30, 596–603. doi: 10.1111/jhn.12451 28168764

[B10] Cohen J. (1988). Statistical Power Analysis for the Behavioral Sciences, 2nd Edn. Abingdon: Taylor and Francis.

[B11] Cornier M. A. Dabelea D. Hernandez T. L. Lindstrom R. C. Steig A. J. Stob N. R. . (2008). The metabolic syndrome. Endocr. Rev. 29, 777–822. doi: 10.1210/er.2008-0024 18971485 PMC5393149

[B12] Cui X. Zhang Y. Jin Y. Wu Z. Gao Y. Su H. . (2025). Association of cardiometabolic index and depressive symptoms with cardiovascular and cerebrovascular diseases in middle-aged and older adults: a national prospective cohort study. J. Affect. Disord. 391:119940. doi: 10.1016/j.jad.2025.119940 40684955

[B13] Edwards M. B. Cunningham G. (2013). Examining the associations of perceived community racism with self-reported physical activity levels and health among older racial minority adults. J. Phys. Act. Health. 10, 932–939. doi: 10.1123/jpah.10.7.932 23132844

[B14] Esgin T. Hersh D. Rowley K. G. Macniven R. Glenister K. Crouch A. . (2021). Physical activity and self-reported metabolic syndrome risk factors in the aboriginal population in Perth, Australia, measured using an adaptation of the global physical activity questionnaire (GPAQ). Int. J. Environ. Res. Public Health. 18:5969. doi: 10.3390/ijerph18115969 34199675 PMC8199758

[B15] Faul F. Erdfelder E. Buchner A. Lang A. G. (2009). Statistical power analyses using G Power 3.1: tests for correlation and regression analyses. Behav. Res. Methods. 41, 1149–1160. doi: 10.3758/BRM.41.4.1149 19897823

[B16] Felitti V. J. Anda R. F. Nordenberg D. Williamson D. F. Spitz A. M. Edwards V. . (2019). Relationship of childhood abuse and household dysfunction to many of the leading causes of death in adults: the adverse childhood experiences (ACE) study. Am. J. Prev. Med. 56:774. doi: 10.1016/j.amepre.2019.04.001 9635069

[B17] Findlay L. C. (2011). Physical activity among First Nations people off reserve, Métis and Inuit. Health Rep. 22, 47–54. 21510589

[B18] Foulds H. J. A. LaFleur J. Johnson S. R. Moore S. McInnes A. Ferguson L. J. (2025). “Community traditions, community kinship, language, and land bring me a lot of joy”: the importance of culture and social support in the health and wellbeing of Métis people. Int. J. Qual. Stud. Health Well-Being 20:2512663. doi: 10.1080/17482631.2025.2512663 40446153 PMC12128144

[B19] Foulds H. J. A. Shubair M. M. Warburton D. E. R. (2013). A review of the cardiometabolic risk experience among Canadian Métis populations. Can. J. Cardiol. 29, 1006–1013. doi: 10.1016/j.cjca.2012.11.029 23465285

[B20] Foulds H. J. A. Warburton D. E. R. (2014). The blood pressure and hypertension experience among North American Indigenous populations. J. Hypertens. 32, 724–734. doi: 10.1097/HJH.0000000000000084 24609208

[B21] Gaudry A. (2009). Métis. In: *The Canadian Encyclopedia*. Historica Canada. Available online at: https://www.thecanadianencyclopedia.ca/en/article/metis (Accessed October 12, 2025).

[B22] Ginn C. Ginn C. W. C. Barnabe C. Dumont/Vaness Bergum D. Gentes J. Tatrallyay P. (2025). How do health, spirituality and well-being intersect in the Métis Nation of Alberta (MNA) Region 3? A Métis-guided, community-based, participatory study. BMJ Open 15:e089503. doi: 10.1136/bmjopen-2024-089503 39842916 PMC11751914

[B23] Hajizadeh M. Hu M. Asada Y. Bombay A. (2021). Explaining the gaps in psychological distress and suicidal behaviours between non-Indigenous and Indigenous adults living off-reserve in Canada: a cross-sectional study. CMAJ Open 9, E215–E223. doi: 10.9778/cmajo.20200177 33688030 PMC8034301

[B24] Heart and Stroke (2025). Helping to Close the Gap in Indigenous Health. Available online at: https://www.heartandstroke.ca/what-we-do/our-impact/helping-to-close-the-gap-in-indigenous-health (Accessed October 12, 2025).

[B25] Henry R. M. A. Ferreira I. Dekker J. M. Nijpels G. Scheffer P. G. Stehouwer C. D. A. . (2009). The metabolic syndrome in elderly individuals is associated with greater muscular, but not elastic arterial stiffness, independent of low-grade inflammation, endothelial dysfunction or insulin resistance—the Hoorn study. J. Hum. Hypertens. 23, 718–727. doi: 10.1038/jhh.2009.8 19279658

[B26] Herrmann S. D. Heumann K. J. Der Ananian C. A. Ainsworth B. E. (2013). Validity and reliability of the global physical activity questionnaire (GPAQ). Meas. Phys. Educ. Exerc. Sci. 17, 221–235. doi: 10.1080/1091367X.2013.805139

[B27] Humphries K. H. Izadnegahdar M. Sedlak T. Saw J. Johnston N. Schenck-Gustafsson K. . (2017). Sex differences in cardiovascular disease – impact on care and outcomes. Front. Neuroendocrinol. 46, 46–70. doi: 10.1016/j.yfrne.2017.04.001 28428055 PMC5506856

[B28] Ironside A. Ferguson L. Katapally T. Johnson S. Moore S. Mainra N. . (2024). Social experiences, social support and residence as determinants of sedentary behaviour among indigenous adults in Saskatchewan: social environment and sedentary lifestyle. Int. J. Indig. Health 19, 1–19. doi: 10.32799/ijih.v19i1.39588

[B29] Ironside A. Ferguson L. J. Katapally T. R. Hedayat L. M. Johnson S. R. Foulds H. J. A. . (2021). Social determinants associated with physical activity among Indigenous adults at the University of Saskatchewan1. Appl. Physiol. Nutr. Metab. 46, 1159–1169. doi: 10.1139/apnm-2020-0781 34236918

[B30] Ji H. Kim A. Ebinger J. E. Niiranen T. J. Claggett B. L. Bairey Merz C. N. . (2020). Sex differences in blood pressure trajectories over the life course. JAMA Cardiol. 5, 255–262. doi: 10.1001/jamacardio.2019.5306 31940010 PMC6990675

[B31] Kessler R. C. Andrews G. Colpe L. J. Hiripi E. Mroczek D. K. Normand S. L. T. . (2002). Short screening scales to monitor population prevalences and trends in non-specific psychological distress. Psychol. Med. 32, 959–976. doi: 10.1017/S0033291702006074 12214795

[B32] Koprich S. Cripps S. Simms A. J. Tsui N. Edwards S. A. Tobin S. W. . (2024). Monitoring cardiovascular disease in Métis citizens across Ontario, 2012–2020. CJC Open 6, 857–867. doi: 10.1016/j.cjco.2024.03.014 39026622 PMC11252518

[B33] Kulak M. J. Los Angeles W. L. Daniels T. E. Mathis K. J. Gobin A. P. Laumann L. E. . (2024). Increased cardiometabolic risk in healthy young adults with early life stress. Psychosom. Med. 86, 72–82. doi: 10.1097/PSY.0000000000001273 38153259 PMC10922275

[B34] Lai H. P. H. Miles R. M. Bredin S. S. D. Kaufman K. L. Chua C. Z. Y. Hare J. . (2019). “With every step, we grow stronger”: the cardiometabolic benefits of an indigenous-led and community-based healthy lifestyle intervention. J. Clin. Med. 8:422. doi: 10.3390/jcm8040422 30934802 PMC6517932

[B35] Leiter L. A. Fitchett D. H. Gilbert R. E. Gupta M. Mancini G. B. J. McFarlane P. A. . (2011). Cardiometabolic risk in Canada: a detailed analysis and position paper by the Cardiometabolic Risk Working Group. Can. J. Cardiol. 27, e1–e33. doi: 10.1016/j.cjca.2010.12.054 21459257

[B36] Lewis M. E. Volpert-Esmond H. I. Deen J. F. Modde E. Warne D. (2021). Stress and cardiometabolic disease risk for indigenous populations throughout the lifespan. Int. J. Environ. Res. Public Health 18:1821. doi: 10.3390/ijerph18041821 33668461 PMC7918141

[B37] Liu J. Young T. K. Zinman B. Harris S. B. Connelly P. W. Hanley A. J. G. . (2006). Lifestyle variables, non-traditional cardiovascular risk factors, and the metabolic syndrome in an Aboriginal Canadian population. Obesity 14, 500–508. doi: 10.1038/oby.2006.65 16648622

[B38] Löwe B. Spitzer R. L. Gräfe K. Kroenke K. Quenter A. Zipfel S. . (2004). Comparative validity of three screening questionnaires for DSM-IV depressive disorders and physicians' diagnoses. J. Affect. Disord. 78, 131–140. doi: 10.1016/S0165-0327(02)00237-9 14706723

[B39] Martens P. J. Bartlett J. G. Prior H. J. Sanguins J. Burchill C. A. Burland E. M. . (2011). What is the comparative health status and associated risk factors for the Metis? A population-based study in Manitoba, Canada. BMC Public Health 11:814. doi: 10.1186/1471-2458-11-814 22011510 PMC3257314

[B40] Marwaha K. (2022). Examining the role of psychosocial stressors in hypertension. J. Prev. Med. Public Health 55, 499–505. doi: 10.3961/jpmph.21.266 36475315 PMC9742403

[B41] McEligot A. J. McMullin J. Pang K. Bone M. Winston S. Ngewa R. . (2010). Diet, psychosocial factors related to diet and exercise, and cardiometabolic conditions in Southern Californian Native Hawaiians. Hawaii Med. J. 69(5 Suppl. 2), 16–20. 20544604 PMC3158438

[B42] McKinley C. E. Ka'apu K. Scarnato J. M. Liddell J. (2020). Cardiovascular health among U.S. indigenous peoples: a holistic and sex-specific systematic review. J. Evid. Based Soc. Work 17, 24–48. doi: 10.1080/26408066.2019.1617817 32133411 PMC7055486

[B43] Parry-Strong A. Gearry R. Merry T. L. Weatherall M. Davies C. Worthington A. . (2023). A high-quality Aotearoa New Zealand dietary pattern adapting a Mediterranean diet for metabolic health: a feasibility study. BMC Nutr. 9:146. doi: 10.1186/s40795-023-00805-x 38066654 PMC10709956

[B44] Phillips-Beck W. Sinclair S. Campbell R. Star L. Cidro J. Wicklow B. . (2019). Early-life origins of disparities in chronic diseases among Indigenous youth: pathways to recovering health disparities from intergenerational trauma. J. Dev. Orig. Health Dis. 10, 115–122. doi: 10.1017/S2040174418000661 30223914

[B45] Powell-Wiley T. M. Baumer Y. Baah F. O. Baez A. S. Farmer N. Mahlobo C. T. . (2022). Social determinants of cardiovascular disease. Circ. Res. 130, 782–799. doi: 10.1161/CIRCRESAHA.121.319811 35239404 PMC8893132

[B46] Priya S. Mardi V. Kapoor S. Kumar A. Saroj U. Dungdung A. . (2024). Association of glycated hemoglobin with acute ischemic stroke in a tertiary care center in a tribal region of Jharkhand. Cureus 16:e58797. doi: 10.7759/cureus.58797 38784369 PMC11112394

[B47] Ramage-Morin P. L. Bougie E. (2017). Family networks and health among Metis aged 45 or older. Health Rep. 28, 12–20. 29261223

[B48] Randall J. Svenson L. Maximova K. Varughese M. Colquhoun A. James A. . (2019). The Burden of Hypertension and Heart Disease Amongst the Métis Nation of Alberta. Available online at: https://albertametis.com/app/uploads/2018/03/Heart-Disease-Report-v4-reduced_size.pdf (Accessed October 12, 2025).

[B49] Reading C. Wien F. (2009). Health Inequalities and Social Determinants of Aboriginal Peoples' Health. Available online at: www.nccah-ccnsa.ca (Accessed October 12, 2025).

[B50] Reading J. (2015). Confronting the growing crisis of cardiovascular disease and heart health among aboriginal peoples in Canada. Can. J. Cardiol. 31, 1077–1080. doi: 10.1016/j.cjca.2015.06.012 26321431

[B51] Reading J. L. (2009). The Crisis of Chronic Disease Among Aboriginal Peoples: A Challenge for Public Health, Population Health and Social Policy. Victoria, BC: University of Victoria. 185 p.

[B52] Satapathy P. Shabil M. Khatib M. N. Ganesan S. Kaur M. Srivastava M. . (2025). Association of cardiometabolic index and risk of stroke: a systematic review and meta-analysis. J. Stroke Cerebrovasc. Dis. 34:108337. doi: 10.1016/j.jstrokecerebrovasdis.2025.108337 40320099

[B53] Sin N. L. (2016). The protective role of positive well-being in cardiovascular disease: review of current evidence, mechanisms, and clinical implications. Curr. Cardiol. Rep. 18:106. doi: 10.1007/s11886-016-0792-z 27612475 PMC5060088

[B54] Statistics Canada (2009). Aboriginal Peoples Survey, 2006. An Overview of the Health of the Métis Population: Fact Sheet. Available online at: https://www150.statcan.gc.ca/n1/en/pub/89-637-x/89-637-x2009006-eng.pdf?st=8ILH4iao (Accessed October 12, 2025).

[B55] Statistics Canada (2021). Projections of the Indigenous Populations and Households in Canada, 2016 to 2041. Available online at: https://www150.statcan.gc.ca/n1/pub/91-552-x/91-552-x2021001-eng.htm (Accessed October 12, 2025).

[B56] Statistics Canada (2022). Indigenous population continues to grow and is much younger than the non-Indigenous population, although the pace of growth has slowed. The Daily 11-001-X, 1–22.

[B57] Statistics Canada (2025). Table 13-10-0394-01 Leading Causes of Death, Total Population, by Age Group. doi: 10.25318/1310039401-eng.

[B58] Tull E. S. Cort M. A. Gwebu E. T. Gwebu K. (2007). Internalized racism is associated with elevated fasting glucose in a sample of adult women but not men in Zimbabwe. Ethn. Dis. 17, 731–735. 18072387

[B59] Wali S. Hiscock E. C. Simard A. Fung N. Ross H. Mashford-Pringle A. . (2024). Learning from our strengths: exploring strategies to support heart health in indigenous communities. CJC Open 6, 849–856. doi: 10.1016/j.cjco.2023.06.005 39026618 PMC11252507

[B60] Whitaker K. M. Buman M. P. Odegaard A. O. Carpenter K. C. Jacobs D. R. Sidney S. . (2018). Sedentary behaviors and cardiometabolic risk: an isotemporal substitution analysis. Am. J. Epidemiol. 187, 181–189. doi: 10.1093/aje/kwx209 28595346 PMC5860012

[B61] Williams D. R. (1999). Race, socioeconomic status, and health the added effects of racism and discrimination. Ann. N. Y. Acad. Sci. 896, 173–188. doi: 10.1111/j.1749-6632.1999.tb08114.x 10681897

[B62] Williams D. R. Yu Y. Jackson J. S. Anderson N. B. (1997). Racial differences in physical and mental health: socio-economic status, stress and discrimination. J. Health Psychol. 2, 335–351. doi: 10.1177/135910539700200305 22013026

